# Chiari I malformation with Klippel-Trenaunay syndrome: case report and review of the literature

**DOI:** 10.1007/s00381-020-04992-x

**Published:** 2021-01-25

**Authors:** Isabel A. Snee, Catherine A. Mazzola, Tatiana Sikorskyj

**Affiliations:** 1 The University of Notre Dame, Notre Dame, IN USA; 2 The New Jersey Pediatric Neuroscience Institute, Morristown, NJ USA

**Keywords:** Klippel-Trenaunay syndrome, Chiari I malformation, Hemihypertrophy, Port-wine stain birthmarks, Tissue and bone overgrowth, Venous malformations

## Abstract

We present a rare case of an 8-year-old male with Klippel-Trenaunay syndrome (KTS) and a Chiari I malformation (CIM). Magnetic resonance imaging (MRI) to investigate facial asymmetry and speech delay at age two revealed CIM with cerebellar tonsils 1.3 cm below the foramen magnum without syringomyelia. The patient underwent a craniectomy and posterior fossa decompression with C1 laminectomy. While gene sequencing determined the patient was negative for the *PIK3CA* gene mutation, the patient’s clinical history strongly suggests KTS. He has hemihypertrophy, leg length discrepancy, hemangiomas and pigmentary mosaicism along the upper and lower extremities, heart murmur, chronic low heart rate, recurrent hip pain, and mild scoliosis. Neurodevelopmental concerns include difficulty reading, attention deficit hyperactivity disorder (ADHD), anxiety, and difficulty running and going downstairs. His most recent MRI shows good decompression at the cervicomedullary junction, global cerebrospinal fluid (CSF) flow, and less peg-like cerebellar tonsils. Also noted were two intravertebral hemangiomas at T5 and T6. While the patient’s speech has improved, there is still difficulty with the expressive language. He still has mild delays, runs slowly, and does not alternate feet when climbing stairs. The patient is being followed by multiple specialists including neurology, hematology-oncology, genetics, orthopedic surgery, and developmental pediatrics.

## Introduction

Klippel-Trenaunay syndrome (KTS) is the rare, congenital, and abnormal development of blood vessels, bones, and soft tissues. With an estimated 1 in 100,000 cases worldwide [1] and 1 in 27,500 newborns [2] affected, there is no preference for race, gender, or hereditary pattern [3, 4]. Features include port-wine stain birthmarks, tissue and bone overgrowth, and venous malformations [5]. Prenatal diagnosis relies on limb hypertrophy and associated varicosities [6]. KTS may be reclassified as Klippel-Trenaunay-Weber syndrome when overlapping with Sturge-Weber syndrome, a rare congenital neurological and skin disorder. Patients may present with lesions, hydrocephalus, vascular malformations, or cerebral calcification [7–10]. With significant clinical overlap with Sturge-Weber syndrome, Parkers-Weber syndrome, and Proteus syndrome, it proves difficult to identify KTS [11]. The Arnold-Chiari I malformation (CIM) describes cerebellar caudal displacement and herniated cerebellar tonsils below the foramen magnum greater than 3 mm in children [12, 13]. Raised intracranial pressure, low intraspinal pressure, hemorrhage or lesions near the foramen magnum or posterior fossa, cerebrospinal fluid (CSF) flow blockage, or insufficient posterior cranial fossa development may cause CIM [13, 14]. Children may present with brainstem dysfunction—sleep apnea or feeding difficulties [15]—, pain, headaches in the occipital or cervical regions after Valsalva movements [12], and scoliosis [12].

## Case Report

**Fig. 1:**
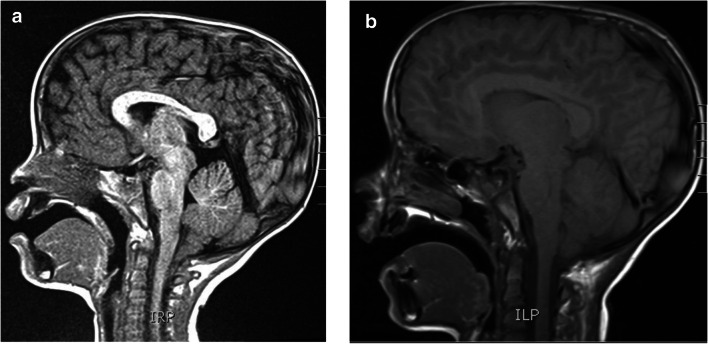
**a** Sagittal view MRI of the patient at 2 months of age. As can be seen above, the cerebellar tonsils protrude into the foramen magnum at approximately 1.3 cm, demonstrating a Chiari I malformation sagittal view MRI of the patient at eight years of age. **b** There is complete resolution of the Chiari I malformation, the formation of more normal cerebellar tonsils, global CSF flow, and overall decompression

We present an 8-year-old male, who at the age of 2 years old had daily headaches and neck pain. An MRI, ordered for facial asymmetry, speech delay, headaches, and neck pain, demonstrated CIM with cerebellar tonsils 1.3 cm below the foramen magnum without syringomyelia (Fig. 1a). The patient eventually underwent a suboccipital craniectomy and C1 laminectomy. A bone-only decompression was done without dural opening or duroplasty. There were no complications.

Past surgical history included tonsillectomy and tympanostomy tube placement for sleep apnea confirmed with polysomnography. Cardiology evaluation at age seven confirmed an innocent heart murmur and low heart rate. His podiatrist prescribed him new lifts for increasing and recurrent hip pain due to leg length discrepancy. His pediatric orthopedist diagnosed him with hemihypertrophy since his right leg and right side of his face are larger than the left side. His pelvic asymmetry induced mild scoliosis of 11°. The patient’s pigmentary mosaicism—with right-sided hyperpigmented streaks on the face, upper, and lower extremities—is monitored by his dermatologist. His spinal hemangiomas are being followed and monitored by pediatric hematology-oncology. Family history was significant for hypertension (HTN) in multiple family members. His maternal grandmother has hyperlipidemia, diabetes, and epilepsy, and is hematologic. His mother has dyslexia, his sister has ADHD and anxiety, and the father has ADHD, dyslexia, and a heart murmur. The sister and father have osteogenesis imperfecta. There were no family members with platybasia, basilar invagination, or syringomyelia. While most KTS patients possess the *PIK3CA* gene mutation, gene sequencing revealed he was negative for this mutation. However, given his clinical history’s consistency with KTS and that other diagnosed KTS patients lack this mutation, KTS was strongly suspected. As noted by his geneticist, the negative result does not exclude a genetic basis for the condition that may be present in him or his family; instead, the results should be interpreted within the contexts of other laboratory results, family history, and his most relevant clinical presentations (Table 1).

**Table 1:** Table of the clinical presentations of KTS with the triad of most common symptoms highlighted for convenience. While not all of these presentations can occur in one patient, the occurrence of one or more should prompt the physician to consider the possibility of KTS, which may or may not be confirmed via genetic testing

Clinical presentation of Klippel-Trenaunay syndrome (the three key clinical diagnostic findings are highlighted below)
Cutaneous capillary malformation (“port-wine stain”)
Variable overgrowth (hypertrophy) of soft tissue and bones, usually in the lower limb (possibly involving feet) and less commonly in trunk and upper extremities
Abnormal veins and/or venous malformations or varicose veins
Lymphatic abnormalities or cysts
Arteriovenous connections
May have RASA1 or PIK3CA mutations
Possible cellulitis or other skin infections
Occasional rectal or vaginal bleeding
Abnormal fatty deposits
Rare disseminated or localized intravascular coagulopathy
Possible blood clots or anemia
Rarely seizures
Possible scoliosis or kyphoscoliosis
Cataracts or glaucoma
Hip dislocation, hip/pelvic asymmetry, or tilt
Rarely Chiari I malformation

A follow-up at age eight, his mother reports that he has difficulty reading at his age level and that he is hyperactive. His pediatrician noted signs of ADHD and anxiety, so he was enrolled in an individualized education program (IEP). To compensate for difficulty walking downstairs and slower-than-normal running, he attended weekly physical therapy during the school year. Brain and spine MRI at age eight showed cervicomedullary decompression, global CSF flow, and resolution of cerebellar tonsillar deformity (Fig. 1b). There were two 1-cm by 1-cm hemangiomas at T5 and T6. With no evidence of vertebral bone collapse, these lesions will be monitored and followed conservatively. The patient communicates in full sentences but has difficulty with expressive language. The mother reports that he still runs slowly and does not alternate feet when climbing stairs. He will return for his annual follow-up next year. In the meantime, he will revisit his specialists and inform us of new or recurring problems.

## Discussion

KTS exhibits port-wine stain birthmarks, tissue and bone overgrowth, and vein malformations, with possible lymphatic abnormalities (Fig. 2) [1]. KTS is rare, with an incidence of 1 in 100,000 [1]. Approximately 63% of affected individuals present with three signs or symptoms, usually involving one lower extremity [16, 17]. Capillary malformations appear flat and range from pale pink to deep maroon [18, 19]. Originating from swollen small blood vessels, these blisters may burst and become infected [19, 20]. Imbalances from bone and tissue overgrowth reduce movement and cause pain, scoliosis, and walking problems [5]. Overgrowth appears more with age, plateauing around the age twelve [20–22]. Vein malformations include painful, swollen, and twisted varicose veins, painful superficial veins, and inflamed clots [5]. Deep vein abnormalities increase deep vein thrombosis risk, possibly leading to a pulmonary embolism [4, 19, 21]. Ancillary symptoms include cataracts, glaucoma, hip dislocation, blood clotting, cellulitis, lymphedema, internal bleeding, acrodactyly, syndactyly, polydactyly, congenital hip dislocation, peripheral neuropathy, metatarsal, and phalangeal agenesis to osteolysis, as well as larger cardiovascular, gastrointestinal, liver, spleen, and genitourinary tract problems [4, 5, 19, 22–25]. Additionally, the moderate psychomotor delay has been described in KTS and may be related to 2q27.3 microdeletion [26].

KTS usually arises from a *PIK3CA* gene mutation, which normally signals for cell growth, division, movement, and survival [19]. However, as mentioned in this case report, KTS has been diagnosed for patients without this genetic mutation, suggesting that more than one gene may be necessary to facilitate the onset and progression of this syndrome. The genetic causes of KTS are poorly understood and genetic mutations are likely responsible for cellular proliferation, angiogenesis, soft tissue hypertrophy, and other cellular abnormalities. It is interesting that many of these abnormal growth patterns are variably distributed in a mosaic or lateralized fashion, even within a given patient. The absence of the PIK3CA mutation, therefore, does not preclude the diagnosis of KTS since capillary malformation, soft tissue hypertrophy, and venous malformations are present in this child. As genetic technologies improve, we will continue to explore any genetic mutations or deletions. Next-generation sequencing (NGS) may in the future allow us to better define the genetic causation for the clinical presentation of KTS in this child. Other etiology theories propose that developmental disorders like embryonic mesodermal tissue malformation interfere with angiogenesis [11, 21]. Others suggest intrauterine trauma, like increased capillary and venular flow without a detectable deep vein obstruction [11, 21, 27]. Intrauterine sympathetic ganglia injury may dilate arteriovenous shunts and precipitate soft tissue hypertrophy and venous enlargement [20, 28]. Chronic venous hypertension from increased arterial flow or deep venous abnormalities may cause nevus, varices, and hypertrophy [17, 20]. Typical conservative treatment includes medical drugs or physical therapy, yet pulsed laser dye therapy, the most common treatment, cosmetically decreases visible capillary malformation [11]. Physiotherapy and compression alleviate most venous malformations, yet extreme cases may need sclerotherapy [11]. With many different treatment pathways, physicians must tailor a specific treatment plan.

There are only three other identified KTS cases with CIM lacking syringomyelia, and these shared similar symptoms to our patient. With no KTS family history, they presented with hemihypertrophy, venous varicosities, and general malformations (two cases further shared hemangiomas, while one shared tonsil asymmetry and scoliosis) [1, 7, 13]. Scoliosis incidence with CIM ranges from 15 to 50% [12], and only 10.5% to 27.3% of those do not have syringomyelia [29]. It is not clear if our patient’s scoliosis was caused by his Chiari I malformation, KTS itself or his leg length discrepancy and pelvic tilt. KTS patients may develop progressive scoliosis from leg length discrepancies, pelvic obliquity or tilt, or truncal asymmetry. Certainly, his scoliosis has not progressed and last year was measured as 11° of thoracic levoscoliosis. Our patient also had psychomotor delay and ADHD. These developmental issues may have been associated with his Chiari I diagnosis or KTS. Chiari type 1 malformation is much more common than KTS and often presents in childhood, and both have been associated with neurodevelopmental problems. Pediatric neurologists and neurological surgeons should be aware of this possible association of C1M and KTS. Additionally, children with C1M and KTS may be predisposed to developing scoliosis with or without syringomyelia, for a variety of reasons. In conclusion, although the presentation of C1M with KTS is rare in children, these children require careful, interdisciplinary care coordination and follow-up.

## Data Availability

Not applicable.

## Abbreviations

ADHD: Attention deficit hyperactivity disorder
CIM: Chiari I malformation
CSF: Cerebrospinal fluid
HTN: Hypertension
KTS: Klippel-Trenaunay syndrome
MRI: Magnetic resonance imaging
